# The Relationship Between Severe Hypertensive Diseases of Pregnancy and Moderate-Severe Bronchopulmonary Dysplasia

**DOI:** 10.21203/rs.3.rs-3373933/v1

**Published:** 2023-09-29

**Authors:** Erica Wymore, Anne Lynch, Jasleen Singh, Tamara Thevarajah, Jennifer Hodges, John Kinsella, Emily Auer, Brandie Wagner

**Affiliations:** University of Colorado School of Medicine; University of Colorado School of Medicine; University of Colorado School of Medicine; University of Colorado School of Medicine; University of Colorado School of Medicine; Colorado School of Public Health

## Abstract

**Objective:**

Determine the association between severe hypertensive disease of pregnancy (HDP) with moderate-severe bronchopulmonary dysplasia (BPD) in preterm infants (< 31 weeks’ gestation).

**Study Design:**

Preterm birth cohort study of 693 mother-infant dyads. Severe HDPwas defined as severe preeclampsia, HELLP syndrome or eclampsia. The outcome was moderate-severe BPD classified at 36 weeks corrected gestational age, based on the NICHD Consensusstatement.

**Results:**

225 (32%) mothers developed severe HDP and 234 (34%) infants hadmoderate-severe BPD. There was an interaction between severe HDP and gestational age (p=0.03). Infants born at earlier gestational ages to mothers with HDP had increased odds for moderate-severe BPD compared to infants of normotensive mothers delivering at the same gestational age. Infants born at later gestational ages to mothers with severe HDP had decreased odds for the outcome.

**Conclusions:**

Severe HDP has a differential effect on the development of moderate-severe BPD based on gestational age.

## INTRODUCTION

Premature very low birth weight infants comprise approximately 1.5% of all U.S. births, yet contribute to more than 50% of the neonatal mortality rate. (1) It is recognized that infants with a low birth weight or gestational age develop severe neonatal complications such as poor neurodevelopmental outcomes, retinopathy of prematurity (ROP), bronchopulmonary dysplasia (BPD), and death. Bronchopulmonary dysplasia, defined as the chronic lung disease of prematurity, was originally described in premature infants with respiratory distress syndrome after birth requiring mechanical ventilation and oxygen therapy. The pathology of the lungs at postmortem examination demonstrated diffuse fibroproliferative lung disease. (2) More recently, researchers have demonstrated a contribution of abnormal pulmonary vascular development to the pathogenesis of BPD. (3–7) Despite major advances in neonatal care and more conservative strategies for mechanical ventilation, up to 40% of premature infants still progress to the development of this chronic lung disease. (8)

Preeclampsia, HELLP (hemolysis, elevated liver enzymes and low platelet count) syndrome, and eclampsia are hypertensive diseases of pregnancy (HDP) associated with abnormal vascular growth within the uteroplacental interface resulting in dysfunctional placentation. (9, 10) Therefore, both BPD and HDP share a common pathogenesis of aberrant vascular development (11–14). For this reason, there is increasing interest in potential links between BPD and HDP. Some investigators have found a relationship between BPD in the infant and HDP in the mother (15–18), while other studies have shown no relationship between BPD and HDP. (19, 20) This may be attributable to the broad clinical spectrum of BPD. Thus, the purpose of our study was to investigate if there is a relationship between HDP and severity of BPD utilizing a defined classification system, in a Colorado cohort of infants born prematurely.

## MATERIALS/SUBJECTS AND METHODS

We conducted a retrospective cohort study using records from a Retinopathy of Prematurity (ROP) registry developed by the Department of Ophthalmology at the University of Colorado School of Medicine. Infants included in the registry delivered at the University of Colorado Hospital between January 1, 2012, through December 31, 2020. The study received approval from the Colorado Multiple Institutional Review Board. The study was performed in accordance with the Declaration of Helsinki. All infants fulfilled the American Academy of Pediatrics 2013 screening criteria for ROP defined as “infants with a birth weight of ≤ 1500 g or gestational age of 30 weeks or less (as defined by the attending neonatologist) and selected infants with a birth weight between 1500 and 2000 g or gestational age of > 30 weeks with an unstable clinical course, including those requiring cardiorespiratory support and who were believed by their attending pediatrician or neonatologist to be at high risk for ROP.”(21)

The ROP Registry is described in detail elsewhere. (22, 23) In brief, the registry contains information on the maternal medical history, pregnancy complications, events during labor and delivery, ROP, and other neonatal outcomes. A dedicated team of trained research assistants performed the perinatal data abstraction retrospectively. The ROP Registry includes Quality Control measures with procedures for automatic variable verification and an annual secondary review of approximately 20% of the records. (22, 23)

The primary exposure was severe HDP (severe preeclampsia, eclampsia, or the HELLP syndrome(24) defined in detail by the American College of Obstetrics and Gynecology 2013 Guidelines. (25) Classification of HDP was independently conducted by A.M.L and T.S.T. by chart review. Uncertain cases were reviewed by J.K.H. and a consensus was reached. These reviewers were masked to the BPD outcome.

The primary outcome of the study was moderate-severe BPD. We used the BPD classification described by the National Institute of Child Health and Human Development (NICHD) consensus panel and adjusted for altitude in Denver, CO to define BPD.(26–28) Infants were defined as having BPD if they required supplemental oxygen at 28 days of life and with formal oxygen reduction testing via pulse oximetry at 36 weeks postconceptional age (PCA), or at discharge, whichever was first. BPD severity was classified as 1) Mild- room air or need for < 26% oxygen or discharged home on supplemental oxygen prior to 36 weeks PCA; 2) Moderate- need for > 26% to < 35% oxygen; or 3) Severe- need for > 35% oxygen and/or need for positive pressure respiratory support, based on oxygen correction at altitude compared to sea level. Documentation of formal oxygen reduction testing by NICU staff was required for BPD classification. Classification of BPD was performed independently and retrospectively by E.M.W. and T.S.T. Any discrepancies were resolved by additional chart review by other study investigators.

Information on maternal demographics, maternal self-designation of race/ethnicity, pregnancy and delivery history were included in the analysis. Adverse outcomes related to preterm birth were also assessed, including severe (grade 3 or 4) intraventricular hemorrhage (IVH)(29), surgical and medical necrotizing enterocolitis (NEC)(30). Other variables examined were small for gestational age (SGA) defined as birth weight < 10th percentile for gestational age described by Fenton preterm growth curve, (31), gestational age at delivery and birth weight. All placentas from pregnancies complicated by preterm birth who deliver at our institution have a pathological examination performed. The diagnosis of histological chorioamnionitis is made using a standard approach. (32)

We excluded from the original cohort (n = 926) infants with: 1) ROP screening criteria by unstable clinical course alone, rather than by gestational age or birth weight criteria, 2) congenital or chromosomal anomalies, 3) a transfer to an outside institution prior to ROP examination, 4) an uncertain BPD classification, and 5) babies from women with gestational hypertension (as the focus of the study was severe forms of HDP). Following exclusions, 693 mother-infant dyads remained in the analytic dataset, with all infants surviving to 36 weeks PCA for their BPD determination. A consort diagram describing exclusion of infants from the analytic dataset is shown in supplementary Fig. 1.

### Statistical Analysis:

Descriptive statistics were calculated using means and standard deviations for continuous variables and percentages for categorical variables. Risk factors were compared across HDP and BPD groups using chi-squared tests and two-sample t-tests for categorical and continuous variables, respectively. The association between severe HDP, gestational age and moderate-severe BPD was evaluated graphically using a loess scatterplot smoother. The multivariable logistic regression model included both an interaction between HDP and gestational age as well as birth weight. Statistical analysis was performed using SAS 9.4 (SAS Institute, Cary, NC).

## RESULTS

In our cohort, 225 (32%) mothers had severe HDP, 234 (34%) had moderate-severe BPD. We show in Table 1 the characteristics of the cohort and the risk factors by severe HDP status. The majority of women were white. The mean gestational age (± SD, range) at delivery was 28 weeks (± 2.0, range = 23 weeks to 30 weeks and 6 days), with 68 (10%) of the entire cohort delivering ≤ 25 weeks.

Women with severe HDP were more likely to be older, have a cesarean delivery, deliver an infant with a birth weight < 10%, and be at a higher gestational age at delivery. We found a significantly lower incidence of treatment with antibiotics in women with severe HDP compared with women who had a preterm birth and remained normotensive. There was no difference in the frequency of the secondary infant outcomes examined between infants born to normotensive women compared to hypertensive women.

We demonstrate in Table 2 cohort characteristics by moderate-severe BPD status. It is noteworthy that there was no significant association between maternal severe HDP and BPD status. Infants who fell into the moderate–severe BPD category were significantly more likely to have a lower gestational age and birth weight at delivery.

**Figure 1:**
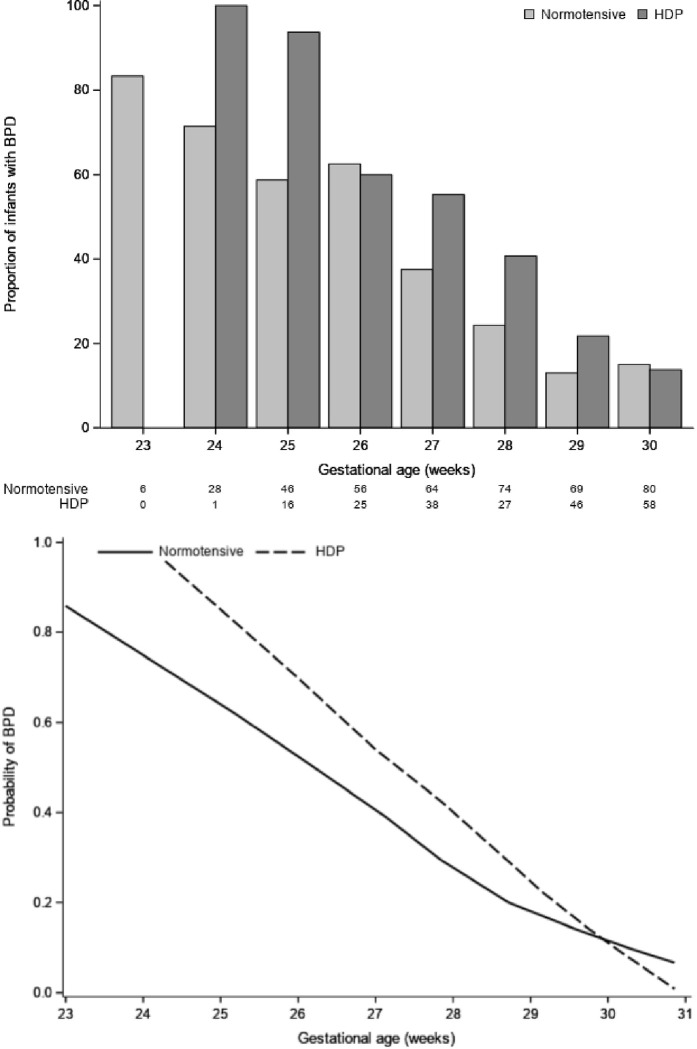
**(a):** Probability of moderate-severe BPD for infants born from normotensive mothers and mothers with HDP by gestational age. The proportion of infants with BPD by gestational age and HDP. The numbers at the bottom correspond to the number of infants in each group. The proportion of those with HDP (darker grey) that develop BPD is higher than those from normotensive mothers. The proportion of babies with BPD decreases with increasing gestational age and the difference between those born from HDP and normotensive mothers also decreases (Top). **(b):**The association between moderate-severe BPD and gestational age by HDP is indicated with a scatterplot smoother (loess curve). The curves indicate that there may be a difference in the slope of the curves between normotensive (solid) and HDP (dashed) that suggests an interaction between HDP and gestational age (bottom).

The association between HDP and the moderate-severe BPD outcome was first inspected graphically (Fig. 1). In this figure, we demonstrate that the risk for moderate-severe BPD decreases with increasing gestational age. Figure 1 also indicates a difference in risk associated with HDP, where infants with lower gestational ages have higher risk of moderate-severe BPD compared to infants from normotensive mothers. This difference becomes less pronounced with higher gestational age. These associations were formally evaluated using a multivariable logistic regression (Table 3). In the logistic regression model, we tested an interaction between HDP and gestational age.

We show in Table 3 the odds ratios associated with lower gestational age by HDP groups estimated from the multivariable logistic regression. For infants from normotensive mothers, the odds of moderate-severe BPD increased by 1.2 (95% CI 1.0–1.5) associated with each 1 week decrease in gestational age. The odds for an infant from a mother with HDP was higher at 1.6 (95% CI 1.3–2.1), p-value for interaction = The interaction indicates that the odds for moderate-severe BPD increases more rapidly with lower GA in infants from mothers with HDP compared to infants from normotensive mothers.

**Figure 2:**
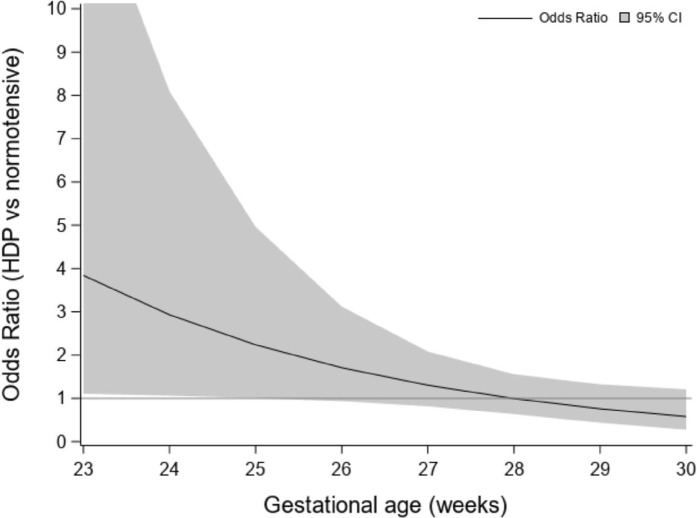
Odds ratio estimates for the risk of moderate-severe BPD among infants of mothers with HDP compared to infants of normotensive mothers across gestational age. Estimates were obtained from the multivariable logistic regression including an interaction between HDP and gestational age. The 95% CI band excludes 1 up to 25 weeks gestational age.

The odds of BPD associated with HDP across gestational age is displayed in Fig. 2. This figure shows increased odds of developing moderate-severe BPD for babies from mothers with HDP only in the lower gestational ages. For example, at 25 weeks gestational age an infant from a mother with HDP has an odds ratio for moderate-severe BPD of 2.2 (95% CI: 1.0–5.0) compared to an infant born to a normotensive mother. However, the increased odds associated with HDP is not observed at 28 weeks GA. The 95% CI excludes values of 1 up to 25 weeks of gestational age suggesting that the increased odds for moderate-severe BPD associated with HDP is most pronounced in babies born at 25 weeks or less. The association with HDP is no longer present when the interaction is excluded from the logistic regression (OR 1.1 (95% CI: 0.7–1.7)).

## DISCUSSION

In this cohort, we investigated the relationship between severe HDP and moderate-severe BPD. We found no association between HDP and moderate-severe BPD in a univariate analysis or after adjusting for GA and birthweight. However, we found an important and critical interaction between gestational age and severe HDP and severity of the BPD outcome. Infants born < 25 weeks gestation to mothers with HDP had twice the odds of developing moderate-severe BPD compared to infants of normotensive mothers. This risk decreased with progressive gestational age.

Several studies have investigated the association of HDP with BPD, defined as oxygen need at 28 days and at 36 weeks post conceptual age, with inconsistent findings. Hansen et al examined a cohort of babies born between 23 and 32 weeks gestation and found that BPD was increased in infants exposed to preeclampsia.(15) Gemmell et al studied an international cohort of 27,000 preterm neonates born between 24 and 28 weeks gestation and found similar results.(18) Other authors have also described this increased association.(16, 17). Of relevance to the results of our study, Razak et al published a systematic review and meta-analysis of pregnancy-induced hypertension and neonatal outcomes. (33) In agreement with the results of our study, these investigators also found an increased odds of BPD in a subgroup analysis of infants < 29 weeks gestation born to preeclamptic mothers, but not for all preterm infants. In contrast, several multi-center studies have not found a relationship between BPD with HDP.(19, 20) Smaller, single center studies have found associations with preeclampsia and BPD, however after adjustment for growth restriction, these associations were no longer significant. (34–36) We suggest the discordant results could be attributed to different sample sizes, wider ranges of gestational ages, different definitions of HDP and/or severity of BPD as well as omission of the potential interaction between gestational age and HDP.

The pathophysiology of BPD is characterized by an arrest of both alveolarization and of pulmonary vascular development, secondary to multiple antenatal and perinatal insults when infants are born at the late canalicular stage of lung growth.(3–7) Preeclampsia also has links with dysregulation of angiogenic function that starts early in pregnancy with an imbalance in circulating angiogenic factors.(37) We hypothesize from the results of our study that altered levels of angiogenic factors may impact pulmonary vascular development in the fetus, in a maturational or gestational age dependent manner. Our findings of differential risk of moderate-severe BPD based on gestational age in infants born to women with HDP suggests that this risk may be attributable to the severity of HDP in earlier gestation affecting lung maturation and subsequent development at a critical stage.

Our study was limited by sample size. It is understood that the risk of BPD increases with lower gestational age, and severity of HDP resulting in premature delivery is also inversely related to gestational age. As such, there is no inherent control group when examining premature deliveries, which is an unresolvable challenge for any such study. Placental vascular abnormalities were not available thus not included in this analysis. However, strengths of our study include strict definitions of HDP and severity of BPD classification, not included in other investigations. We utilized the NICHD classification for BPD severity, to limit the variability in the clinical spectrum of BPD seen in previous studies. Our cohort’s outcome definition included a rigorously defined oxygen reduction test to determine BPD severity. Another major strength of our analysis is the inclusion of the important interaction between gestational age and HDP, addressing this recognized inter-relationship.

We found that severe HDP has a differential effect on the development of moderate-severe BPD based on gestational age among preterm infants at risk for ROP. The presence of this effect may explain why certain studies have found an association while others have not. These findings suggest that biologic factors specific to severe HDP occurring earlier in pregnancy contribute to the development of moderate-severe BPD. Further investigation in larger carefully characterized cohorts is warranted to study these biologic mechanisms between maternal pathophysiology and subsequent neonatal pulmonary vascular development among preterm infants.

## Figures and Tables

**Table 1:** Cohort Characteristics and Risk Factors by Severe Hypertensive Disease (HDP) of Pregnancy Status

Risk Factors	Total n = 693	HDP n = 225	Normotensive n = 468	P Value
Maternal Age, Mean (SD)	29 (6)	31 (6)	29 (6)	<0.01
Maternal Race, n (%) White	433 (62%)	155 (69%)	278 (59%)	0.03
Black/African American	103 (15%)	24 (11 %)	79 (17%)	
Other	157 (23%)	46 (20%)	111 (24%)	
Hispanic Ethnicity, n (%)	195 (28%)	58 (26%)	137 (29%)	0.34
Multiple Gestation, n (%)	141 (20%)	41 (18%)	100 (21%)	0.34
Tobacco Use in Pregnancy, n (%)	81 (12%)	20 (9%)	61 (13%)	0.11
Histologic Chorioamnionitis, n (%)	276 (40%)	11 (5%)	265 (57%)	<0.01
Antenatal Corticosteroids, n (%)	667 (96%)	215 (96%)	452 (97%)	0.51
Antenatal Antibiotics, n (%)	338 (56%)	39 (17%)	349 (75%)	<0.01
Cesarean Delivery, n (%)	464 (67%)	204 (91%)	260 (56%)	<0.01
Male Sex, n (%)	359 (52%)	108 (48%)	251 (54%)	0.16
Gestational Age, Weeks (SD)	28.0 (2.0)	28.3 (1.7)	27.8 (2.0)	<0.01
Birth Weight, grams (SD)	1053 (330)	963 (286)	1096 (340)	<0.01
SGA (BW < 10%)	58 (8%)	39 (17%)	19 (4%)	<0.01

**Abbreviations:** HDP = Severe hypertensive disease of pregnancy (preeclampsia, HELLP and eclampsia); SGA = small for gestational age; BW = birth weight

**Table 2:** Selected Cohort Characteristics by Category of Bronchopulmonary Dysplasia (BPD)

Risk Factors	Mod-Severe BPD n = 234	No/Mild BPD n = 459	P Value
Maternal Age, Mean (SD)	30 (7)	29 (6)	0.65
Maternal Race, n (%)			0.06
White	158 (68%)	275 (60%)	
Black/African American	25 (11 %)	78 (17%)	
Other	51 (22%)	106 (23%)	
Hispanic Ethnicity, n (%)	61 (26%)	134 (71%)	0.39
Multiple Gestation, n (%)	41 (18%)	100 (22%)	0.19
Tobacco Use in Pregnancy, n (%)	30 (13%)	51 (11 %)	0.51
Histologic Chorioamnionitis, n (%)	94 (40%)	182 (40%)	0.77
Maternal Severe HDP n (%)	81 (35%)	144 (31%)	0.39
Antenatal Corticosteroids, n (%)	222 (95%)	445 (97%)	0.17
Antenatal Antibiotics, n (%)	137 (59%)	251 (55%)	0.33
Cesarean Delivery, n (%)	167 (71%)	297 (65%)	0.08
Male Sex, n (%)	123 (53%)	236 (53%)	0.77
Gestational Age Weeks (SD)	26.7 (1.8)	28.6 (1.7)	<0.01
Birth Weight, grams (SD)	842 (249)	1161 (313)	<0.01
SGA (birth weight < 10%)	34 (15%)	24 (5%)	<0.01

**Abbreviations:** BPD = bronchopulmonary dysplasia. Please see Table 1 for other abbreviations

**Table 3:** Odds Ratios for Moderate-Severe BPD outcome (N = 693) *

	OR	95% CI	P Value
Birthweight (decrease in 100 g)	1.30	1.17–1.45	<0.01
GA in infants from normotensive mothers (decrease in 1 week)	1.23	1.04–1.47	0.02
GA in infants from mothers with HDP (decrease in 1 week)	1.62	1.28–2.06	<0.01
HDP at mean GA	0.99	0.64–1.56	0.98

* Multivariable logistic regression analysis includes birthweight and an interaction between HDP and gestational age.
